# Crystallization and Assembly-Driven Nanostructures for Energy, Electronics, Environment, and Emerging Applications

**DOI:** 10.3390/nano13040637

**Published:** 2023-02-06

**Authors:** Jihua Chen

**Affiliations:** Center for Nanophase Materials Sciences, Oak Ridge National Laboratory, Oak Ridge, TN 37830, USA; chenj1@ornl.gov

This manuscript has been authored by UT-Battelle, LLC, under Contract No. DEAC05-00OR22725 with the U.S. Department of Energy. The United States Government and the publisher, by accepting the article for publication, acknowledges that the United States Government retains a nonexclusive, paid-up, irrevocable, worldwide license to publish or reproduce the published form of this manuscript, or allow others to do so, for United States Government purposes. DOE will provide public access to these results of federally sponsored research in accordance with the DOE Public Access Plan (http://energy.gov/downloads/doe-public-access-plan, accessed on 30 March 2022).

Self-assembly and crystallite control are key processes that determine the performance and applications of numerous materials and nanomaterials found in nature and society. To further break down the areas of interest, the discussion will be on (i) synthetic polymers, (ii) biological materials, and (iii) inorganic crystallites, respectively.

(i) For synthetic polymers and soft complexes, (semi-) crystalline or self-assembled nanostructures play important roles in technologies such as sensors, actuators, solar cells, transistors, supercapacitors, new battery designs, solid electrolytes, biomimetic designs, and smart materials as well as structural components [1]. For example, polymer-based battery materials and organic semiconductors are often crystalline or semicrystalline. Nanocomposites and block copolymers often rely on various intramolecular or intermolecular forces to form nanostructures and nanopatterns. Reference [1] reviewed nanostructure analysis for synthetic soft materials, highlighting the current status of machine learning techniques in the field.

(ii) Self-assembly in biological materials can be well-suited for medical applications. Materials found in nature, such as spider silk, shells, and bones, often rely on crystallization or assembly mechanism to achieve extraordinary performance. For example, extracellular vesicles (EV) have attracted attention as new drug carriers due to their unique structural properties and self-assembly [2]. Reference [2] designed a technical strategy with Cytochalasin B treatment at hypotonic conditions to optimize assembly and allow increased drug loading capacity of EV for cancer treatment.

(iii) Controlling the crystalline components of inorganic materials attracts an intense scientific interest, in efforts to achieve optimization in crystallography, crystallization process (including dynamics), hierarchical morphology, nanostructure, nanopattern, or assembly-driven interfaces and composites. These changes largely contribute to the further enhancements in mechanical, ionic, electronic, and other functional behaviors of current-generation inorganic nanomaterials [3,4,5]. For example, Ultrasonic nanocrystalline surface modification represents a unique approach for mechanical impact-based surface deformation [3]. It has the potential towards gradient nanostructured surface layers, which can be impactful in the future generation of transportation, energy, medical, and chemical industries. Cryomilling and Spark Plasma Sintering are among other important approaches for enhancing the crystallites and properties of pure [4] and composite inorganics [5].
